# Predialysis Care Trajectories of Patients With ESKD Starting Dialysis in Emergency in France

**DOI:** 10.1016/j.ekir.2020.10.026

**Published:** 2020-10-31

**Authors:** Maxime Raffray, Cécile Vigneau, Cécile Couchoud, Sahar Bayat

**Affiliations:** 1 University of Rennes, French School of Public Health (EHESP), Pharmaco-epidemiology and health Services Research, Rennes, France; 2 University of Rennes, CHU Rennes, Inserm, EHESP, Irset (Institut de recherche en santé, environnement et travail), Rennes, France; 3 REIN Registry, Biomedecine Agency, Saint-Denis-La-Plaine, France

**Keywords:** big data, care trajectories, emergency dialysis start, end-stage kidney disease, health care consumption

## Abstract

**Introduction:**

Emergency dialysis start (EDS) is frequent for patients with chronic kidney disease (CKD). To improve CKD management, new trajectory-based care policies are currently being introduced both in France and in the United States. This study describes the different types of predialysis care trajectories and factors associated with EDS.

**Methods:**

Adults patients who started dialysis in France in 2015 were included. Individual clinical and health care consumption data were retrieved from the French national end-stage kidney disease (ESKD) registry (Renal Epidemiology and Information Network [REIN]) and the French National Health Data system (SNDS), respectively. Hierarchical Clustering on Principal Component was used to identify groups of patients with the same health care consumption profile during the 2 years before dialysis start. Logistic regression analysis was used to identify factors associated with EDS.

**Results:**

Among the 8856 patients included in the analysis, 2681 (30.3%) had EDS. The Hierarchical Clustering on Principal Component identified six types of predialysis care trajectories in which EDS rate ranged from 13.8% to 61.8%. After adjustment for the patients’ characteristics, less frequent or lack of follow-up with a nephrologist was associated with higher risk of EDS (odds ratio [OR]: 1.32; 95% confidence interval [CI]: 1.17–1.50 and OR: 1.83; 95% CI: 1.58–2.12), but not follow-up with a general practitioner.

**Conclusions:**

The care trajectories during the 2 years before dialysis start were heterogeneous and patients with a lesser or lack of follow-up with a nephrologist were more likely to start dialysis in emergency, regardless of the frequency of follow-up by a general practitioner (GP). New CKD policies should include actions to strengthen CKD screening and referral to nephrologists.

## Graphical abstract

**Figure:**
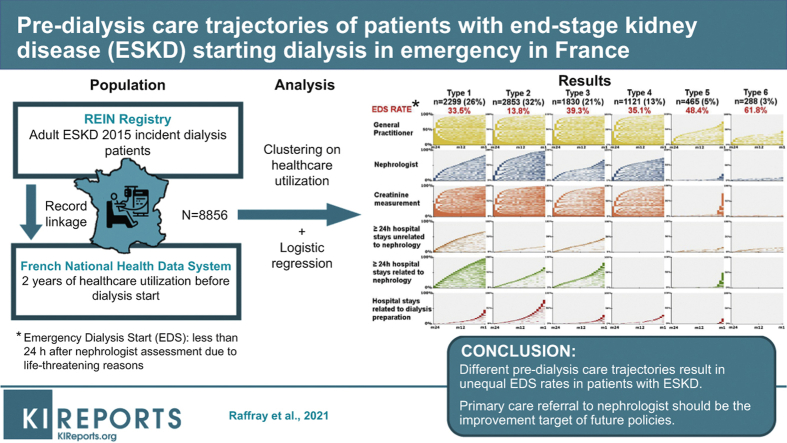

See Commentary on Page 7

Starting maintenance dialysis in a planned manner remains a challenge for many patients with ESKD.^1^ Besides the question of when exactly a patient known to nephrology services should start dialysis, planned dialysis start (PDS) implies many preliminary organizational and medical steps, including the initial screening and diagnosis of CKD, disease monitoring, referral to a nephrologist, therapeutic education of the patient, and preparation for renal replacement therapy. In this context, the coordinated work of GP and nephrologist is considered a key factor to delay CKD progression and also to ensure that patients start renal replacement therapy in the best possible conditions.^2^^,^^3^

In many countries, guidelines on CKD management have been published,^4^ including in France.^5^ Yet, despite the existence of these recommendations, past studies have reported that 20% to 60% of patients start dialysis in an unplanned manner or in emergency.^1^^,^^6^ There is no consensus definition of EDS, some studies refers to it as “starting in a life threatening situation,” some as “a first dialysis without a permanent access device in place.”^1^ In France and in this study, EDS is defined as a first dialysis session started within 24 hours after a nephrologist assessment due to life-threatening reasons. The EDS rate has not decreased since the publication of such guidelines in France: in 2018, 30% of incident dialyzed patients started dialysis in emergency.^7^

In addition to its high rate, EDS can have negative consequences on the postdialysis care trajectory compared with PDS. Higher risk of morbidity and mortality^1^^,^^8^, ^9^, ^10^, ^11^ and a lower quality of life^12^^,^^13^ have been reported. High EDS rates can also be considered a marker of inefficient CKD management. Recent health care policies have been unveiled in France and the United States to improve CKD care with financial incentives to promote the prevention of disease progression and dialysis start in optimal conditions.^14^^,^^15^ Previous studies have found that patients starting dialysis in emergency have more comorbidities,^8^^,^^16^ and that a late referral to a nephrologist is associated with EDS.^17^^,^^18^ Yet, early referral does not exclude EDS, and other factors at the organizational and patient level could be implicated.^19^, ^20^, ^21^, ^22^

The care trajectory that precedes dialysis start has not been investigated yet, particularly how ambulatory and hospital care change over time depending on the patients’ comorbidities. Therefore, this study used patient clinical and health care consumption data to 1) identify and describe types of care trajectory during the last 2 years before dialysis start; 2) compare the predialysis care trajectories in patients who had EDS and PDS; and 3) identify factors associated with EDS.

## Methods

### Study Population and Data Sources

All ≥18-year-old patients who started maintenance dialysis in France in 2015 were identified in REIN database, the French national registry for ESKD. The REIN registry records all patients who start chronic renal replacement therapy in France.^23^ It collects patient data at dialysis start and records specific events upon occurrence (i.e., transplantation and death). However, it does not contain information on health care consumption before renal replacement therapy initiation. To get that information, the 2015 incident patients identified in REIN were matched to the SNDS database using an iterative deterministic record linkage procedure.^24^ The SNDS database covers 99% of the population in France and collects data on 1) the reimbursement of ambulatory health care consumption (e.g., medical consultations, biological tests, and drug delivery) and 2) hospital activity (i.e., inpatient and outpatient stays, diagnoses, procedures, and length of stay).^25^ Unmatched patients from the REIN registry with the SNDS database and patients without information on the dialysis start condition (EDS or PDS) were excluded.

### Outcome

EDS was the event of interest, as recorded in the REIN registry as a first dialysis session started within 24 hours after a nephrologist assessment due to life-threatening reasons, including acute pulmonary edema, severe hyperkalemia or acidosis, uremic confusion, or pericarditis.

### Covariates

The REIN registry data included patients’ demographic features (age and sex) and several comorbidities: cardiovascular diseases, diabetes, chronic respiratory disease, active malignancy, hepatic diseases, and mobility (walking without help, need of assistance, or totally dependent). Additionally, nephropathies were reviewed and classified by three nephrologists (including co-authors C.V and C.C) on a clinical knowledge basis in three groups according to their progression rate: slowly progressive, acute, and uncertain/variable nephropathy (Supplementary Table S1).

Using SNDS data, eight variables of interest were constructed to describe the care trajectory during the last 2 years before dialysis.

First, ambulatory care included: 1) consultations with a GP; and 2) consultation with a nephrologist; and 3) creatinine measurement. The number of consultations and creatinine measurement per semester (or 6-month period) was retrieved. Then, patients were assigned to different categories of follow-up: “at least 1 consultation/measurement every semester,” “less than 1 consultation/measurement every semester,” or “absent.” All consultations that occurred during a hospital stay were excluded. The semester was chosen as time unit to assess the follow-up regularity to facilitate comparison with the CKD management guidelines in which the semester is the most common time unit.^5^

Second, the hospital care (excluding the dialysis start hospitalization) included: 4) number of hospital stays shorter than 24 hours; 5) number of hospital stays longer than 24 hours with a nephrology-related diagnosis (Supplementary Table S2); and 6) number of hospital stays longer than 24 hours related to any other diagnosis. The *International Classification of Diseases, Tenth Revision* was used. The last two variables concerned the 7) total number of days spent in hospital by each patient; and 8) type of preparatory care for dialysis in the 2 years before dialysis. Preparatory care was classified into three groups: creation of an arteriovenous fistula creation or peritoneal dialysis catheter placement; no fistula (or peritoneal dialysis catheter) but at least 1 hospital stay with a diagnosis related to dialysis preparation care which include echography of the upper limbs and tunneled central venous catheter placement; or no preparation.

### Statistical Analyses

The characteristics of the patients with EDS and PDS at dialysis start and their predialysis care trajectory were reported using percentages for categorical variables and medians with interquartile ranges for continuous variables. Because of the exhaustive nature of our recruitment, descriptive data were not compared between the EDS and PDS groups using statistical tests.

A multiple correspondence analysis was first performed using the eight categorical care trajectory variables. This step allows reducing the data noise and cluster stabilization. This was followed by Hierarchical Clustering on Principal Component of the multiple correspondence analysis results using Ward’s hierarchical agglomerative clustering method.^26^

Next, logistic regression analysis was used to examine the association between EDS and the patients’ characteristics at dialysis start, and the variables describing the predialysis care trajectory. Missing data were handled using the multiple imputation by chained equation procedure with 10 iterations to create five imputed datasets to stabilize the results.^27^ The pooled ORs and 95% CIs were reported. All statistical analyses were performed using R, version 3.6.

## Results

### Study Population

**Figure 1:**
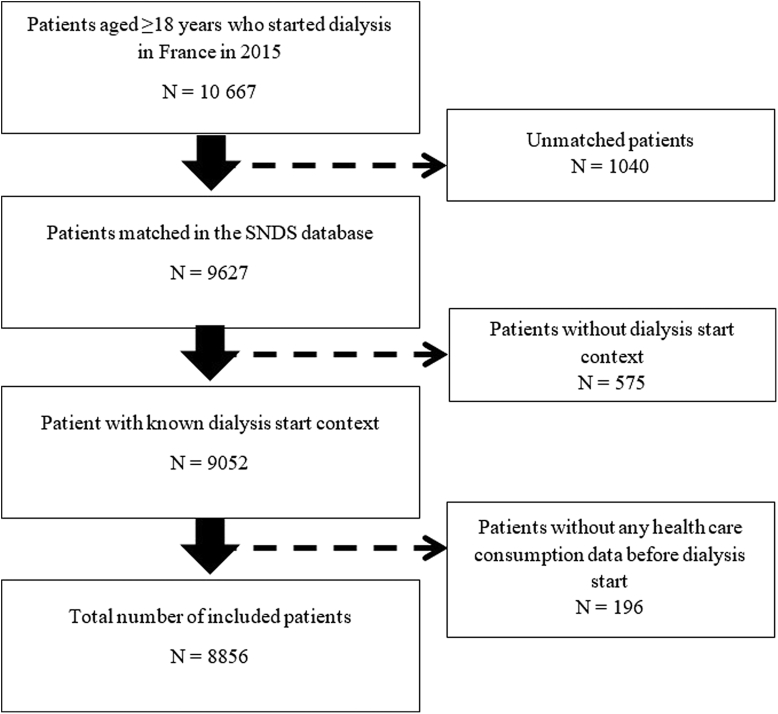
Flowchart of patient selection. SNDS, French National Health Data system.

In 2015, 10,667 patients started dialysis in France, among whom 90.2% were matched in the SNDS database using the deterministic record linkage method (Figure 1). Patients without information on the dialysis start context (EDS or PDS) were excluded as well as 196 patients without any health care consumption data in the 2 years before dialysis start. In total, 8856 patients were included, among whom 2681 (30.3%) had EDS.

### Types of Predialysis Care Trajectories

**Figure 2:**
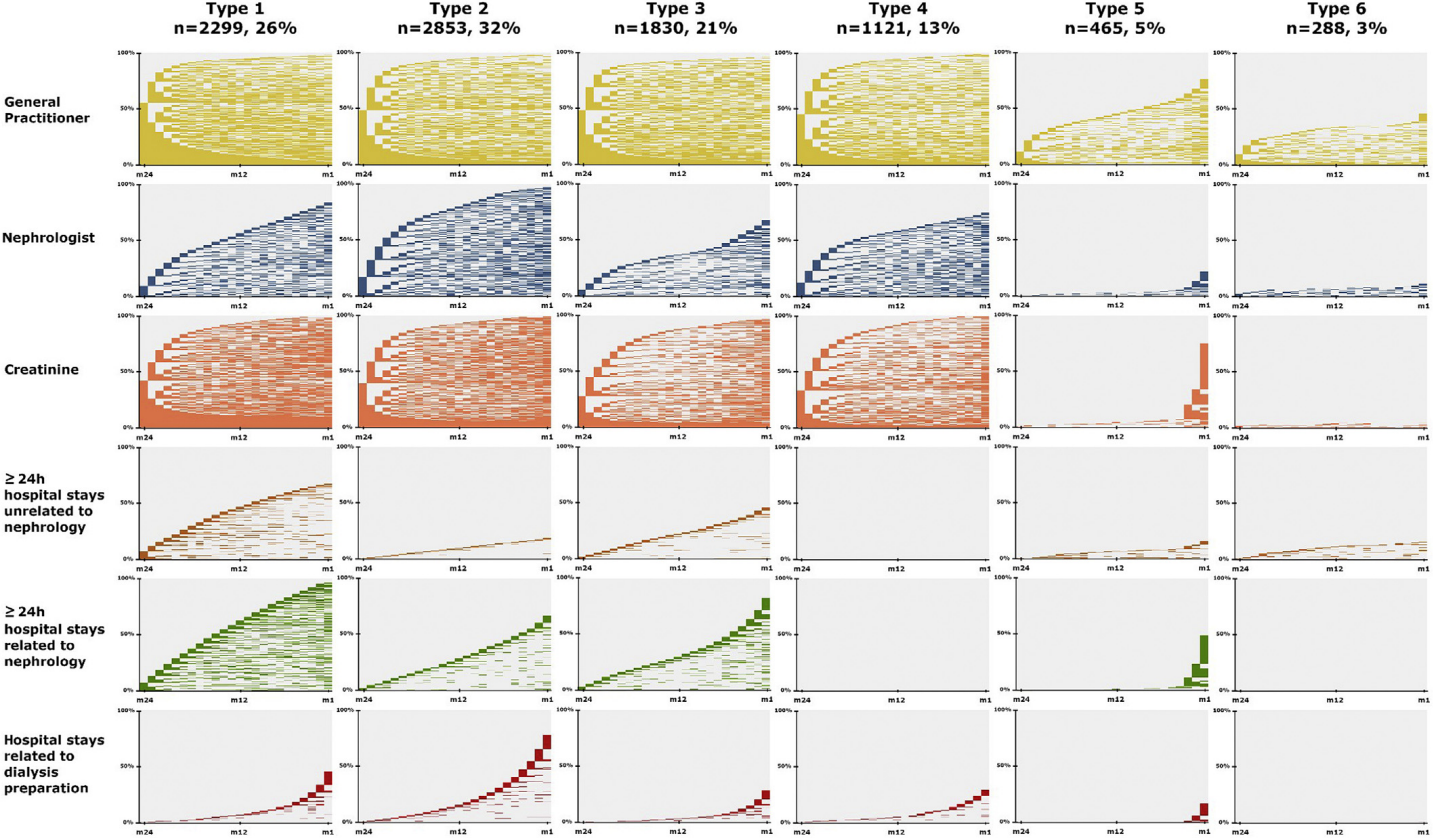
Types of predialysis trajectories in the 2 years before dialysis start identified using the Multiple Correspondence Analysis (MCA) and Hierarchical Clustering on Principal Component (HCPC) methods. There are six clusters of patients identified by MCA and HCPC. For each cluster, the different components of the predialysis care trajectory are shown (list on the left side). The x-axis represents the time (in months) during the last 2 years before dialysis start (month 24 to month 1). The y-axis represents the percentage of patients in that cluster. For each month and for each patient, the event of interest (e.g., consultation with a general practitioner, with a nephrologist, hospitalization, and creatinine monitoring) was either present (color) or absent (blank). Patients with the event of interest present are displayed first at month 24; then, the rest of the individual trajectories are displayed.

Upon clustering, the 8856 patients included were divided into six groups sharing a similar pattern of health care consumption during the 2 years before dialysis start (i.e., six care trajectory types) (Figure 2). Tables 1 and 2 summarize the predialysis health care consumption and the characteristics at dialysis start of the patients in the six care trajectory groups. EDS rates ranged from 13.8% (type 2) to 61.8% (type 6).

**Table 1:** Health care consumption in the 2 years before dialysis start by the incident dialysis patients for 2015 according to their care trajectory type

	Type of predialysis care trajectory						
	1	2	3	4	5	6	Total population
	(n = 2299)	(n = 2853)	(n = 1830)	(n = 1121)	(n = 465)	(n = 288)	(N = 8856)
**Emergency dialysis start rate, %**	**33.5**	**13.8**	**39.3**	**35.1**	**48.4**	**61.8**	**30.3**
Consultations with a GP							
At least 4 times per semester	944 (41.1)	643 (22.5)	424 (23.2)	216 (19.3)	9 (1.9)	8 (2.8)	2244 (25.3)
At least once per semester	946 (41.1)	1577 (55.3)	1072 (58.6)	611 (54.5)	91 (19.6)	43 (14.9)	4340 (49.0)
Less than once every semester	362 (15.7)	586 (20.5)	264 (14.4)	282 (25.2)	257 (55.3)	81 (28.1)	1832 (20.7)
No follow-up	47 (2.0)	47 (1.6)	70 (3.8)	12 (1.1)	108 (23.2)	156 (54.2)	440 (5.0)
Consultations with a nephrologist							
At least once every semester	1125 (48.9)	2388 (83.7)	296 (16.2)	563 (50.2)	12 (2.6)	19 (6.6)	4403 (49.7)
Less than once every semester	797 (34.7)	390 (13.7)	953 (52.1)	272 (24.3)	91 (19.6)	14 (4.9)	2517 (28.4)
No follow-up	377 (16.4)	75 (2.6)	581 (31.7)	286 (25.5)	362 (77.8)	255 (88.5)	1936 (21.9)
Creatinine monitoring							
At least 1 test per semester	2235 (97.2)	2770 (97.1)	1122 (61.3)	853 (76.1)	31 (6.7)	7 (2.4)	7018 (79.2)
Gap of at least 1 semester after 1 test	64 (2.8)	82 (2.9)	700 (38.3)	265 (23.6)	13 (2.8)	6 (2.1)	1130 (12.8)
Only ≥1 test in the last 3 mo before dialysis	0 (0)	1 (0.0)	8 (0.4)	2 (0.2)	421 (90.5)	0 (0)	432 (4.9)
No testing	0 (0)	0 (0)	0 (0)	1 (0.1)	0 (0)	275 (95.5)	276 (3.1)
Number of hospital stays (<24 h)^a^							
0	838 (36.5)	1378 (48.3)	1237 (67.6)	734 (65.5)	360 (77.4)	236 (81.9)	4783 (54.0)
1	500 (21.7)	730 (25.6)	392 (21.4)	230 (20.5)	54 (11.6)	31 (10.8)	1937 (21.9)
≥2	961 (41.8)	745 (26.1)	201 (11.0)	157 (14.0)	51 (11.0)	21 (7.3)	2136 (24.1)
Number of hospital stays (≥24 h) unrelated to nephrology							
0	750 (32.6)	2298 (80.5)	982 (53.7)	1121 (100)	389 (83.7)	243 (84.4)	5783 (65.3)
1	570 (24.8)	460 (16.1)	650 (35.5)	0 (0)	49 (10.5)	16 (5.6)	1745 (19.7)
≥2	979 (42.6)	95 (3.3)	198 (10.8)	0 (0)	27 (5.8)	29 (10.1)	1328 (15.0)
Number of hospital stays (≥24h) related to nephrology a							
0	87 (3.8)	937 (32.8)	319 (17.4)	1121 (100)	235 (50.5)	288 (100)	2987 (33.7)
1	112 (4.9)	1225 (42.9)	715 (39.1)	0 (0)	176 (37.8)	0 (0)	2228 (25.2)
≥2	2100 (91.3)	691 (24.2)	796 (43.5)	0 (0)	54 (11.6)	0 (0)	3641 (41.1)
Days spent in hospital							
0	0 (0)	0 (0)	0 (0)	1121 (100)	172 (37.0)	243 (84.4)	1522 (17.2)
≥1 and ≤10	28 (1.2)	2172 (76.1)	603 (33.0)	0 (0)	188 (40.4)	32 (11.1)	3035 (34.3)
≥11 and ≤31	671 (29.2)	669 (23.4)	1107 (60.5)	0 (0)	74 (15.9)	11 (3.8)	2533 (28.6)
>31	1600 (69.6)	12 (0.4)	120 (6.6)	0 (0)	31 (6.7)	2 (0.7)	1766 (19.9)
RRT preparation type							
Fistula or peritoneal dialysis catheter	1096 (47.7)	2415 (84.6)	554 (30.3)	310 (27.7)	76 (16.3)	0 (0)	4451 (50.3)
Other dialysis preparation care	303 (13.2)	93 (3.3)	77 (4.2)	59 (5.3)	36 (7.7)	2 (0.7)	570 (6.4)
No preparation	900 (39.1)	345 (12.1)	1199 (65.5)	752 (67.1)	353 (75.9)	286 (99.3)	3835 (43.3)

GP, general practitioner; RRT, renal replacement therapy.

Values are n (%) unless otherwise noted.

a Excluding hospital stays for dialysis preparation care;

**Table 2:** Patient characteristics at dialysis start according to their care trajectory type

	Type of predialysis care trajectory						
	1	2	3	4	5	6	Total population
	(n = 2299)	(n = 2853)	(n = 1830)	(n = 1121)	(n = 465)	(n = 288)	(N = 8856)
**Emergency dialysis start, %**	**33.5**	**13.8**	**39.3**	**35.1**	**48.4**	**61.8**	**30.3**
eGFR at dialysis start (ml/min per 1.73 m^2^), median [Q1–Q3]	9.1 [6.8–12.4]	8.1 [6.3–10.3]	8.0 [5.8–10.9]	7.4 [5.4–9.6]	6.1 [4.2–8.7]	6.1 [3.8–8.7]	8.1 [6.0–10.7]
Age, yr							
18–44	111 (4.8)	244 (8.6)	105 (5.7)	78 (7.0)	124 (26.7)	74 (25.7)	736 (8.3)
45–59	296 (12.9)	490 (17.2)	219 (12.0)	190 (16.9)	117 (25.2)	76 (26.4)	1388 (15.7)
60–74	823 (35.8)	1013 (35.5)	693 (37.9)	412 (36.8)	138 (29.7)	79 (27.4)	3158 (35.7)
≥75	1069 (46.5)	1106 (38.8)	813 (44.4)	441 (39.3)	86 (18.5)	59 (20.5)	3574 (40.4)
Men	1432 (62.3)	1816 (63.7)	1205 (65.8)	751 (67.0)	304 (65.4)	187 (64.9)	5695 (64.3)
Nephropathy type							
Slowly progressive nephropathy	1518 (66.0)	2113 (74.1)	1182 (64.6)	764 (68.2)	233 (50.1)	153 (53.1)	5963 (67.3)
Acute nephropathy	297 (12.9)	244 (8.6)	256 (14.0)	131 (11.7)	98 (21.1)	50 (17.4)	1076 (12.1)
Variable/uncertain progression	484 (21.1)	496 (17.4)	392 (21.4)	226 (20.2)	134 (28.8)	85 (29.5)	1817 (20.5)
Number of cardiovascular diseases^a^							
0	623 (27.1)	1526 (53.5)	692 (37.8)	622 (55.5)	309 (66.5)	171 (59.4)	3943 (44.5)
1	585 (25.4)	674 (23.6)	491 (26.8)	258 (23.0)	85 (18.3)	65 (22.6)	2158 (24.4)
2	513 (22.3)	384 (13.5)	357 (19.5)	136 (12.1)	45 (9.7)	22 (7.6)	1457 (16.5)
≥3	578 (25.1)	269 (9.4)	290 (15.8)	105 (9.4)	26 (5.6)	30 (10.4)	1298 (14.7)
Diabetes	1288 (56.0)	1207 (42.3)	868 (47.4)	429 (38.3)	103 (22.2)	91 (31.6)	3986 (45.0)
Missing data	16 (0.7)	8 (0.3)	9 (0.5)	4 (0.4)	0 (0)	3 (1.0)	40 (0.5)
Chronic respiratory disease	471 (20.5)	290 (10.2)	265 (14.5)	111 (9.9)	31 (6.7)	24 (8.3)	1192 (13.5)
Missing data	68 (3.0)	81 (2.8)	56 (3.1)	32 (2.9)	10 (2.2)	13 (4.5)	260 (2.9)
Active malignancy	356 (15.5)	191 (6.7)	216 (11.8)	119 (10.6)	41 (8.8)	19 (6.6)	942 (10.6)
Missing data	53 (2.3)	75 (2.6)	45 (2.5)	23 (2.1)	10 (2.2)	13 (4.5)	219 (2.5)
Mobility							
Walk without help	1575 (68.5)	2396 (84.0)	1342 (73.3)	877 (78.2)	389 (83.7)	217 (75.3)	6796 (76.7)
Need assistance	368 (16.0)	189 (6.6)	228 (12.5)	111 (9.9)	39 (8.4)	28 (9.7)	963 (10.9)
Totally dependent	155 (6.7)	67 (2.3)	100 (5.5)	34 (3.0)	10 (2.2)	13 (4.5)	379 (4.3)
Missing	201 (8.7)	201 (7.0)	160 (8.7)	99 (8.8)	27 (5.8)	30 (10.4)	718 (8.1)

eGFR, estimated glomerular filtration rate.

Values are n (%) unless otherwise stated.

a Congestive heart failure, coronary disease, arrhythmia, aortic aneurysm, arteritis of lower limbs, stroke, or transient ischemic attack.

#### Type 1

Type 1 care trajectory (n = 2299, 26%) included patients with a frequent and regular follow-up by a GP (41.1% saw a GP at least four times per semester) and regular creatinine monitoring. These patients were frequently hospitalized: 91.3% had two or more hospital stays related to nephrology care. Compared with the whole population, these patients were older (46.5% were older than 75 years) and had more frequently comorbidities: 25.1% had at least three cardiovascular diseases. The EDS rate was 33.5%.

#### Type 2

Type 2 care trajectory (n = 2853, 32%) was characterized by a frequent and regular ambulatory follow-up (83.7% saw a nephrologist at least once per semester). Patients spent a short amount of time hospitalized (76.1% spent between 1 and 10 days in hospitalization), and 80.5% had no hospital stay unrelated to nephrology care. Most of them (84.6%) underwent arteriovenous fistula or peritoneal dialysis catheter procedures. Comorbidities were less frequent in this group (53.5% of them had no cardiovascular disease). The EDS rate was 13.8%.

#### Type 3

Type 3 care trajectory (n = 1830, 21%) was characterized by higher rates of less frequent follow-up with a nephrologist (52.1% saw a nephrologist less than once every semester) and creatinine monitoring (38.3% had a gap of at least 1 semester after 1 test). Moreover, only 30.3% of patients in this group underwent arteriovenous fistula or peritoneal dialysis catheter procedures. The EDS rate was 39.3%.

#### Type 4

Type 4 care trajectory (n = 1121, 13%) included mostly patients with regular follow-up by a GP and a nephrologist, and without hospital stays longer than 24 hours. Among them, 55.5% did not have any cardiovascular disease. The EDS rate was 35.1%.

#### Type 5

Type 5 care trajectory (n = 465, 5%) included many patients with less frequent (55.3%) or absent follow-up (23.2%) by a GP. Most of them did not see a nephrologist (77.8%), or only in the last months before dialysis. Creatinine monitoring was late for 90.5% of them, and arteriovenous fistula or peritoneal dialysis catheter procedures were performed only for 16.3%. These patients were younger (52% were aged between 18 and 60 years) and with lower frequency of comorbidities (only 22.2% had diabetes). Acute nephropathy was more frequent in this group (28.8%). The EDS rate was 48.4%.

#### Type 6

The majority of patients in the type 6 care trajectory (n = 288, 3%) did not see a GP or a nephrologist (54.2% and 88.5%, respectively) and did not have any creatinine monitoring (95.5%) before dialysis start. Additionally, none underwent arteriovenous fistula or peritoneal dialysis catheter procedures before dialysis start. These patients were younger, and 29.5% had a nephropathy with a variable or uncertain progression. The EDS rate was 61.8%.

### Comparison of the Predialysis Care Trajectories in the EDS and PDS Groups: Ambulatory Follow-Up by GP and Nephrologist

**Figure 3:**
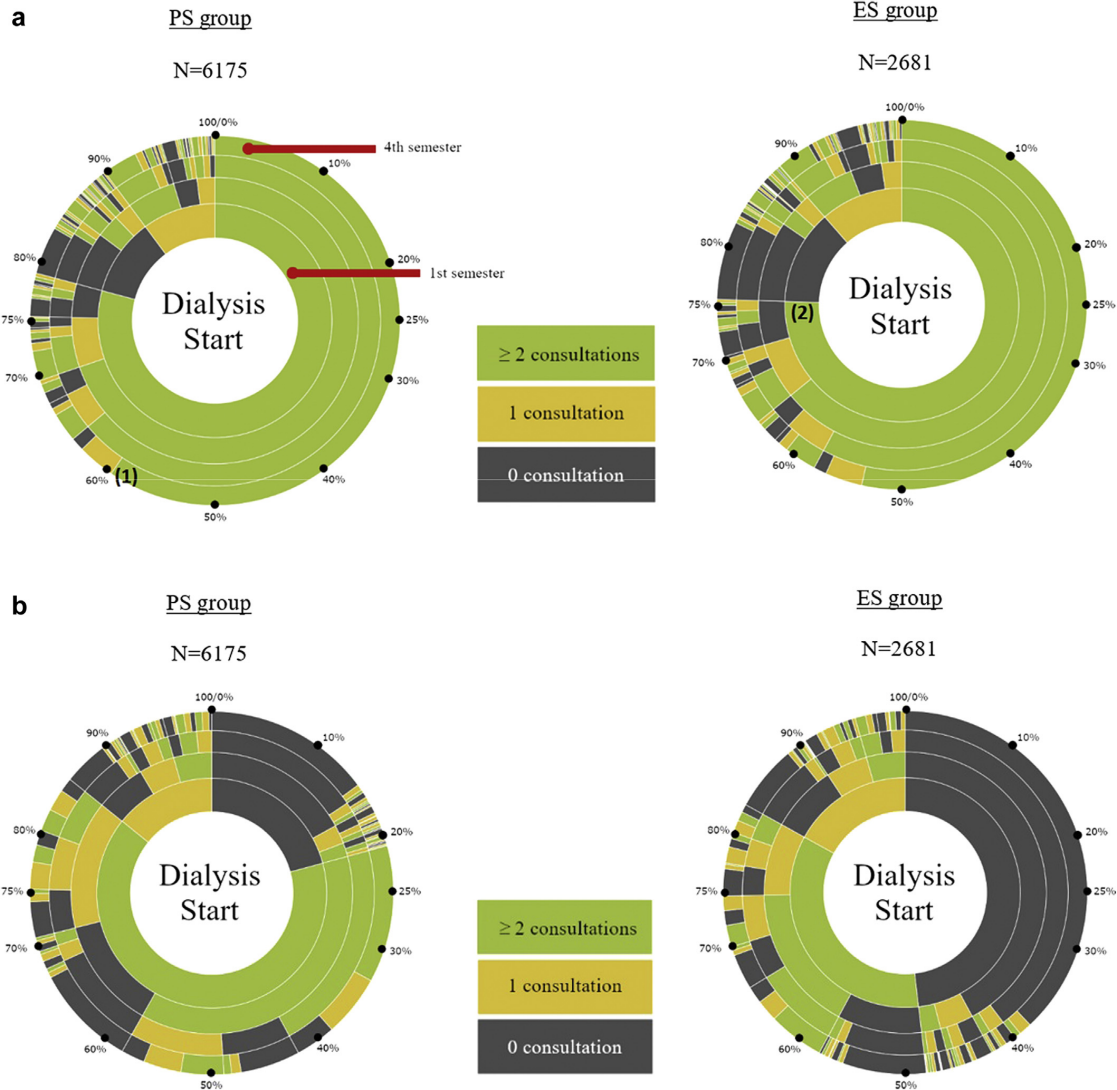
(a) Number of consultations per semester with a general practitioner (GP) in the 2 years before dialysis start and (b) number of consultations per semester with a nephrologist in the 2 years before dialysis start for each patient (1 radius = 1 patient) in the ES and PS groups. Represented are the times from dialysis start (at the center) to 2 years before it (main circle). The period of 2 years is divided into 4 circles, each representing a period of 6 months (or semester). The number of consultations with a GP (a) and a nephrologist (b) for each patient and semester have been retrieved and colored accordingly. The percentages displayed are cumulative. Reading example*:* (1) Among the 2681 patients who started dialysis in emergency, 75% saw the GP at least 2 times in the last 6 months (semester) before dialysis start. (2) Among the 6175 patients who started dialysis in a planned manner, 60% saw the GP at least 2 times every 6 months period (semester) before dialysis start.

The description of the predialysis care trajectories in the EDS and PDS groups is presented in Table 3. Analysis of each patient’s consultation frequency per semester during the 2 years before dialysis start showed that, overall, follow-up with a GP was frequent throughout the entire period and similar between in the EDS and PDS groups. Specifically, 70% and 65% of patients in the PDS and EDS groups, respectively, saw a GP at least twice per semester in the last year before dialysis start (Figure 3A). Conversely, the frequency of consultations with a nephrologist was very different between groups (Figure 3B): 14.9% and 38% of patients in the PDS and EDS groups, respectively, did not see a nephrologist in the 2 years before dialysis start. For most patients followed by a nephrologist, the consultation frequency was twice per semester.

**Table 3:** Ambulatory and hospital care in the 2 years before dialysis start

	Patients with PDS	Patients with EDS	Total
	N = 6175 (69.7%)	N = 2681 (30.3%)	(N = 8856)
Consultations with a GP			
Median (IQR)	14 (8–24)	14 (7–23)	14 (8–23)
At least 4 times per semester	1600 (25.9)	644 (24.0)	2244 (25.3)
At least once per semester	3086 (50.0)	1254 (46.8)	4340 (49.0)
Less than once every semester	1232 (20.0)	600 (22.4)	1832 (20.7)
No follow-up	257 (4.2)	183 (6.8)	440 (5.0)
Consultations with a nephrologist			
Median (IQR)	6 (2–9)	2 (0–5)	5 (1–8)
At least once per semester	3540 (57.3)	863 (32.2)	4403 (49.7)
Less than once every semester	1717 (27.8)	800 (29.8)	2517 (28.4)
No follow-up	918 (14.9)	1018 (38.0)	1936 (21.9)
Creatinine measurement			
At least 1 test per semester	5180 (83.9)	1838 (68.6)	7018 (79.2)
Gap of at least 1 semester after 1 test	670 (10.9)	460 (17.2)	1130 (12.8)
Only ≥1 test in the last 3 mo before dialysis	219 (3.5)	213 (7.9)	432 (4.9)
No testing	106 (1.7)	170 (6.3)	276 (3.1)
At least 1 hospital stay	5751 (93.1)	2229 (83.1)	7980 (90.1)
Median number of hospital stays (IQR)	4.00 (2–6)	3.00 (2–6)	4.00 (2–6)
Median duration of hospital stays, d (IQR)	2.80 (1.2–5.5)	4.20 (2–7.9)	3.00 (1.4–6.2)
Number of hospital stays (<24 h)^a^			
0	3197 (51.8)	1586 (59.2)	4783 (54.0)
1	1401 (22.7)	536 (20.0)	1937 (21.9)
≥2	1577 (25.5)	559 (20.9)	2136 (24.1)
Number of hospital stays (≥24 h) unrelated to nephrology			
0	4160 (67.4)	1623 (60.5)	5783 (65.3)
1	1194 (19.3)	551 (20.6)	1745 (19.7)
≥2	821 (13.3)	507 (18.9)	1328 (15.0)
Days spent in hospital			
0	884 (14.3)	638 (23.8)	1522 (17.2)
≥ 1 and ≤10	2352 (38.1)	683 (25.5)	3035 (34.3)
≥11 and ≤31	1813 (29.4)	720 (26.9)	2533 (28.6)
>31	1126 (18.2)	640 (23.9)	1766 (19.9)
RRT preparation type			
Fistula or peritoneal dialysis catheter	3853 (62.4)	598 (22.3)	4451 (50.3)
Other dialysis preparation care	330 (5.3)	240 (9.0)	570 (6.4)
No preparation	1992 (32.3)	1843 (68.7)	3835 (43.3)

GP, general practitioner; IQR, interquartile range; RRT, renal replacement therapy.

Values are presented as n (%) or mean (IQR).

a Excluding hospital stays for dialysis preparation care.

### Factors Associated With EDS

Comparison of the patient characteristics in the two groups (EDS and PDS) at dialysis initiation (Table 4) indicated that age and sex were comparable between groups. The percentage of patients with acute nephropathy was higher in the EDS than PDS group (15.8% versus 10.6%).

**Table 4:** Characteristics at dialysis initiation of the incident patients in France (2015) according to the dialysis initiation context (N = 8856)

	Patients with PDS	Patients with EDS
	n = 6175 (69.7%)	n = 2681 (30.3%)
Men	3933 (63.7)	1762 (65.7)
Age, yr		
Median (IQR)	71.3 (60.6–80.1)	70.9 (60.6–80.3)
18–44	486 (7.9)	250 (9.3)
45–59	988 (16.0)	400 (14.9)
60–74	2199 (35.6)	959 (35.8)
≥75	2502 (40.5)	1072 (40.0)
Nephropathy type		
Slowly progressive nephropathy	4327 (70.1)	1636 (61.0)
Acute nephropathy	653 (10.6)	423 (15.8)
Variable/uncertain progression	1195 (19.4)	622 (23.2)
Serum albumin <30 g/l	866 (14.0)	691 (25.8)
Missing data	771 (12.5)	399 (14.9)
Hemoglobin <10 g/dl	3018 (48.9)	1742 (65.0)
Missing data	206 (3.3)	125 (4.7)
Number of cardiovascular diseases^a^		
0	2922 (47.3)	1021 (38.1)
1	1515 (24.5)	643 (24.0)
2	955 (15.5)	502 (18.7)
≥3	783 (12.7)	515 (19.2)
Diabetes	2727 (44.2)	1259 (47.0)
Missing data	29 (0.5)	11 (0.4)
Chronic respiratory disease	754 (12.2)	438 (16.3)
Missing data	169 (2.7)	91 (3.4)
Active malignancy	583 (9.4)	359 (13.4)
Missing data	136 (2.2)	83 (3.1)
Hepatic disease	151 (2.4)	79 (2.9)
Missing data	149 (2.4)	81 (3.0)
Mobility		
Totally dependent	193 (3.1)	186 (6.9)
Need assistance	571 (9.2)	392 (14.6)
Walk without help	4956 (80.3)	1840 (68.6)
Missing	455 (7.4)	263 (9.8)

EDS, emergency dialysis start; PDS, planned dialysis start.

Values shown are n (%) or median (IQR).

a Congestive heart failure, coronary disease, arrhythmia, aortic aneurysm, arteritis of lower limbs, stroke, or transient ischemic attack.

**Figure 4:**
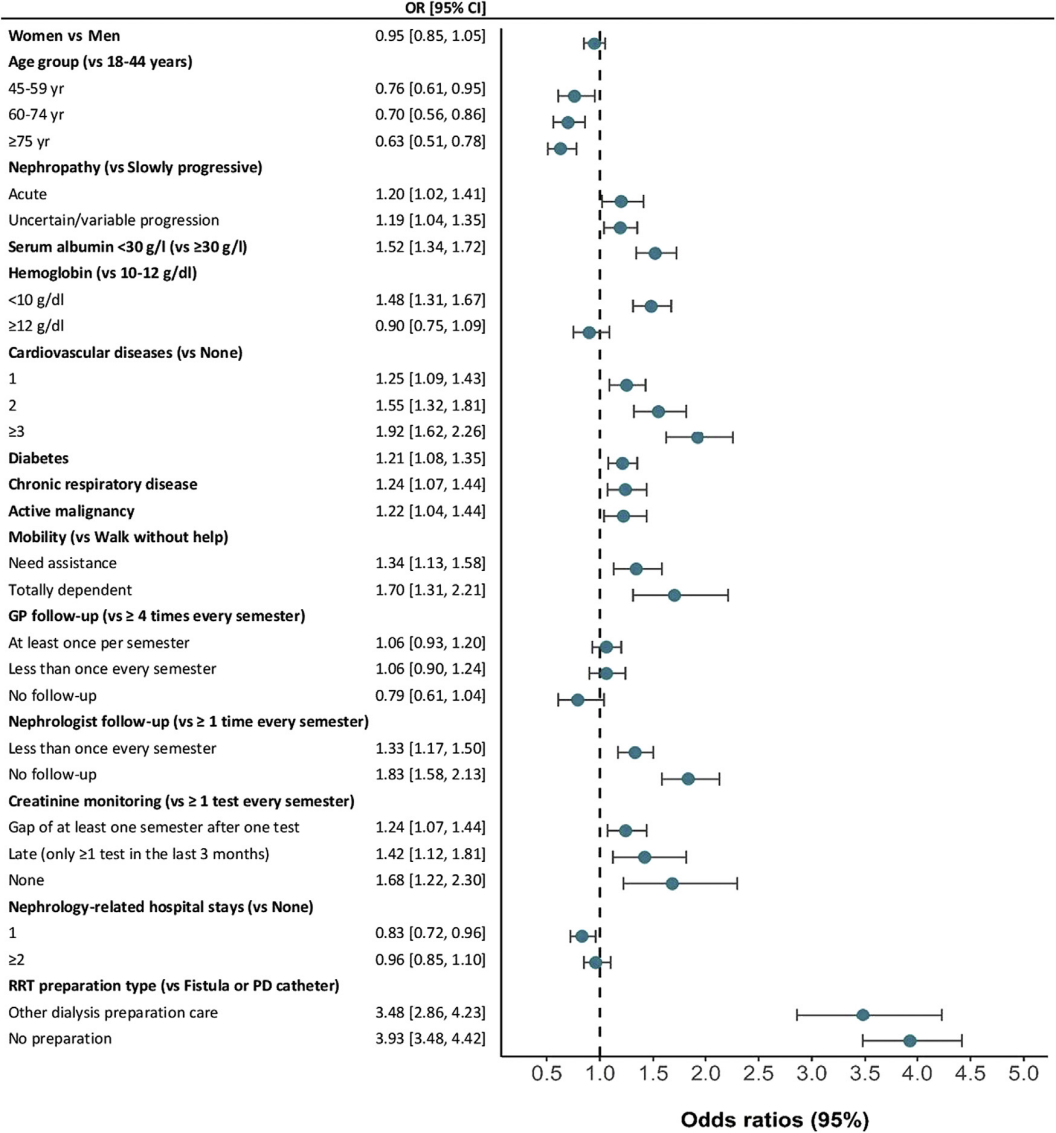
Association of emergency dialysis start with patient characteristics at dialysis initiation and care trajectory using adjusted logistic regression analysis. CI, confidence interval; GP, general practitioner; OR, odds ratio; PD, peritoneal dialysis; RRT, renal replacement therapy.

The complete results of the logistic regression analyses are presented in supplementary material (Supplementary Table S3). The results of the adjusted analysis (Figure 4) indicated that, compared with patients younger than 45 years, the risk of EDS was significantly lower in each age group of patients older than 45 years (OR: 0.63; 95% CI: 0.51–0.78 for ≥75-year-old patients). Compared with patients with slowly progressive nephropathy, the risk of EDS was higher in patients with acute nephropathy (OR: 1.20; 95% CI: 1.03–1.41) and in patients with uncertain progressive nephropathy (OR: 1.19, 95% CI: 1.05–1.35). The risk of EDS progressively increased in patients with 1 or more cardiovascular diseases (OR: 1.92; 95% CI: 1.63–2.27 for patients with three or more cardiovascular diseases). Diabetes (OR: 1.21; 95% CI: 1.09–1.35), chronic respiratory disease (OR: 1.23; 95% CI: 1.06–1.43), and active malignancy (OR: 1.22: 95% CI: 1.04–1.43) were other comorbidities significantly associated with higher risk of EDS.

In the last 2 years before dialysis start, follow-up by a GP was not associated with EDS. Conversely, a less frequent (less than once every semester) and absence of follow-up by a nephrologist were independent risk factors of EDS compared with frequent follow-up (at least once per semester) (OR: 1.32; 95% CI: 1.17–1.50 and OR: 1.83; 95% CI: 1.58–2.12, respectively). Compared with regular creatinine monitoring (at least once per semester), late (at least once in the last 3 months before dialysis start) and absence of monitoring were associated with higher risk of EDS (OR: 1.43; 95% CI: 1.13–1.82 and OR: 1.68; 95% CI: 1.23–2.31, respectively). Finally, the risk of EDS was higher in patients without any record of fistula or peritoneal dialysis catheter procedures compared with patients with records (OR: 3.92; 95% CI: 3.48–4.42).

## Discussion

This is the first study that uses individual clinical and health care consumption data at the national level to describe the predialysis care trajectories and the factors associated with EDS. It shows that the predialysis care trajectories were heterogeneous and that the risk of EDS was higher in patients with less frequent or absent nephrology care in the 2 years before dialysis start, regardless of the follow-up by a GP, which was frequent and regular for most patients.

The EDS rate varied greatly, from 13.8% to 61.8%, in the six predialysis care trajectories. Some predialysis care trajectory types were easily explained by the patients’ characteristics. For instance, high use of hospital care related and unrelated to nephrology care was common in older patients and patients with many comorbidities (type 1). Many comorbidities (diabetes, cardiovascular diseases, active malignancy, chronic respiratory disease, and limited mobility) were risk factors of EDS, in agreement with findings from previous studies.^8^^,^^16^ Patients seen by nephrologists have been highlighted as the most medically complex when compared with patients seen by other subspecialties physicians.^28^ How exactly each comorbidity leads to EDS is unclear; however, our study suggests that to prevent EDS, the care and management of this medical complexity should be considered when producing new care policies. Conversely, trajectories characterized by late or very low health care use, but also high EDS rates (type 5 and 6), were shared by younger patients. We can hypothesize that due to their younger age and lower comorbidity level, they did not visit regularly their GP, if at all. These patients could represent a small group of EDS cases that are very difficult to reduce in addition to patients with acute renal failure who will not recover. Although acute progressive nephropathy is more frequent in the EDS group, 61% of patients in this group had a slowly progressive kidney disease.

Nevertheless, almost 50% of patients in the types 5 and 6 care trajectories were older than 60 years, which raises questions about the high proportion of late and lack of creatinine monitoring and nephrology follow-up. Most of the type 3 patients had a regular follow-up with a GP, but had a less frequent creatinine measurement and were often addressed late to a nephrologist. These findings question the coordination of care and calls for the development of actions targeted to GPs for improving CKD screening and timely referral. Additionally, the absence of association between follow-up by a GP and EDS in our adjusted analysis questions the relevance of the widely advocated model of collaboration between GP and nephrologist, particularly during the period close to dialysis start.^29^ However, most of the patients who were followed-up by a nephrologist were probably initially referred by a GP. That crucial work from the GP might have happened years before the 2 years studied here. The type 2 care trajectory seems to be the ideal trajectory to prevent EDS, as suggested by its lowest percentage of patients with EDS (13.8%). Regular consultations with a nephrologist and creatinine monitoring (at least once per semester) during the 2 years before dialysis start, as well as dialysis preparation (arteriovenous fistula or peritoneal dialysis catheter) were associated with a lower risk of EDS after adjustment for patients’ characteristics. Although age and comorbidity burden were comparable in patients with types 2 and 4 trajectories, their care trajectories were very different, particularly regarding preparation for dialysis. This suggests that other factors explain and shape the care trajectory, and subsequently the risk of EDS.

### Strengths

Compared with previous studies that investigated EDS,^16^^,^^20^, ^21^, ^22^^,^^30^, ^31^, ^32^, ^33^, ^34^ our study has several strengths. It took advantage of the linkage of clinical and health care data at the patient level. The REIN registry data allowed the inclusion of nearly all patients with ESKD in France who started dialysis in 2015. Thanks to the data from a nationwide health care database (SNDS), the predialysis care trajectories could be investigated in its broad definition by analyzing together the ambulatory and hospital care with both public and private health care services and facilities.

### Limits

Although the combination of clinical and health care data brings robustness to the study, it did not allow measuring of some important factors, such as the practices of GPs and nephrologists^35^ and the patients’ views and decisions concerning their CKD management and also their health literacy (i.e., their capacity to take these decisions).^36^, ^37^, ^38^, ^39^, ^40^ Moreover, because of the lack of information in the REIN registry and of reliable data in the SNDS database, individual socioeconomic status could not be studied. This calls for complementary approaches and additional investigations on the role and interactions of socioeconomic status and health literacy with care trajectory and EDS. Additionally, the results of the creatinine lab tests were not available; therefore, the estimated glomerular filtration rate was not assessed in the 2 years before dialysis start, which is important to assess more precisely the primary kidney disease course.^41^ Furthermore, in a universal health care system such as in France, geographical accessibility to health professionals could also be a factor that hinders access to specialists and may explain low use and delayed care.^42^

### Perspectives

Studying the care trajectories of patients with CKD is becoming increasingly relevant as new health care policies are introduced aiming at improving CKD prevention and management, including the reduction of EDS. In the United States, the Centers for Medicare and Medicaid services and its Innovation Center recently unveiled new payment models regarding CKD and ESKD care to be experimented during the coming years.^14^ One of these payment models (Kidney Care First) proposes a fixed payment to nephrology practices on a per-patient basis for managing the care of patients with stages 4 and 5 CKD. Similarly, in France, a type of bundled payment for the management of patients with CKD targeted to health care providers has been introduced in late 2019.^15^ Facilities will receive 1 annual payment per patient for a minimum of 1 consultation with nephrologist, dietitian, and coordination nurse. Although the two health care systems are different in some aspects, this trend of moving from the reimbursement of individual health care consumption towards a care trajectory–based system requires a comprehensive understanding of what exactly are the care trajectories of these patients. This study gives some insights into the predialysis trajectories, how they differ, and what that factors are that are associated with EDS. It suggests that CKD screening and referral to nephrologists are essential targets for CKD care improvement and EDS reduction. As such, emerging health care policies should strengthen the primary care screening of CKD and improve coordination with nephrologists. This includes the communication of a clear delineation of the role and responsibility between the GP and the nephrologist.^43^ In a recent systematic review of international CKD management guidelines, only one (Belgium Center for Evidence-Based Medicine) explicitly described that the role of GP should be the detection and monitoring of CKD, detection of complications, and treatment of cardiovascular risk.^4^ While most guidelines recommend the referral to a nephrologist when estimated glomerular filtration rate decreases to less than 30 ml/min per 1.73 m^2^ (or stage 4), in France, the recommended cut off is higher, at 45 ml/min per 1.73 m^2^ (or stage 3B). Such earlier timing could prove to be effective to slow the progression of CKD.^44^ Moreover, the referral to the nephrologist could be hindered by the apprehension that once the patients referred to the specialist, the GP might lose contact with them. Our study suggests otherwise and shows a continuity of care that should be communicated.

Finally, the blind spots due to the limitations of clinical and health care consumption databases should be addressed with complementary qualitative approaches, specifically through the patients’ perspectives and the health care professionals’ practices.

## Disclosure

All the authors declared no competing interests.

## Supplementary Material

Supplementary File (PDF)

**Table S1.** Classification of the primary kidney diseases in the REIN registry according to their progression.

**Table S2.** Diagnostic codes from the International Classification of Diseases, Tenth Revision (ICD-10) used to identify nephrology-related hospital stays excluding preparatory care for dialysis.

**Table S3.** Factors associated with EDS; results from the unadjusted and adjusted analyses performed using the imputed data, N = 8856.
