# Supplementary material for: Predialysis Care Trajectories of Patients With ESKD Starting Dialysis in Emergency in France
Source: Kidney Int Rep. 2020 Oct 31;6(1):156–67. doi: 10.1016/j.ekir.2020.10.026 (PMC7785414; doi:10.1016/j.ekir.2020.10.026)
Supplement: Supplementary File (PDF) [file mmc1.pdf]

Supplemental material

Table of content Supplemental material

Supplemental Table 1: Classification of the primary kidney diseases in the REIN registry according to their progression

Supplemental Table 2: Diagnostic codes from the International Classification of Diseases, Tenth Revision (ICD-10) used to identify nephrology-related hospital stays excluding preparatory care for dialysis,

Supplemental Table 3: Factors associated with EDS, results from the unadjusted and adjusted analyses performed using the imputed data, N=8856

Supplemental Table 1: Classification of the primary kidney diseases in the REIN registry according to their progression

**Acute nephropathy (including CKD exacerbation/flare)**	**Slowly progressive nephropathy**	**Uncertain/Variable progression**
Amyloidosis	Kidney agenesis, hypoplasia, dysplasia	Glomerulonephritis without histological examination
Extra-capillary glomerulonephritis	Morphological abnormality	Glomerulonephritis with histological diagnosis
Cryoglobulinemia	Diabetes	Secondary glomerular nephritis
Wegener’s granulomatosis	Glomerulonephritis with FSGS	Unknown
Acute kidney injury	Membranous glomerulonephritis	Vascular nephropathy from other cause
Other systemic disease	Membranoproliferative glomerulonephritis	Vascular nephropathy, unprecise cause
Myeloma	Kidney infection	Toxic nephropathy
Ischemic nephropathy, cholesterol emboli syndrome	Hereditary kidney disease	
Lupus nephropathy	Tubulointerstitial nephritis due to neurological bladder	
Vascular nephropathy due to malignant hypertension	Tubulointerstitial nephritis secondary to lithiasis	
Traumatic loss of kidney	Tubulointerstitial nephritis due to acquired obstructive uropathy	
Schönlein-Henoch purpura	Tubulointerstitial nephritis due to congenital obstructive uropathy	
Systemic scleroderma	Other tubulointerstitial nephritis	
Goodpasture’s syndrome	Nephrocalcinosis	
Thrombotic microangiopathy	CKD due to vesicoureteral reflux	
	Hereditary nephropathy with deafness	
	Vascular nephropathy due to hypertension	
	IgA nephropathy	
	Primary oxalosis	
	Cystic disease	
	Polycystic kidney	
	Renal tuberculosis	
	Tubulopathies (Dent, Lowe, Barter…)	
	Renal or urinary tract cancer	

Supplemental Table 2: Diagnostic codes from the International Classification of Diseases, Tenth Revision (ICD-10) used to identify nephrology-related hospital stays excluding preparatory care for dialysis,

**Diagnosis code**	Description
N00-N08	Glomerular diseases
N10-N16	Renal tubulo-interstitial diseases
N17-N19	Renal failure
N20-N23	Urolithiasis
N25-N29	Other disorders of kidney and ureter

Supplemental Table 3: Factors associated with EDS, results from the unadjusted and adjusted analyses performed using the imputed data*, N=8856

	Unadjusted				Adjusted			
	**OR**	**2.5 %**	**97.5 %**	**p**	**OR**	**2.5 %**	**97.5 %**	**p**
**Sex**								
Men	1.00				1.00			
Women	0.91	0.83	1.01	0.07	0.95	0.85	1.05	0.32
**Age**								
18-44	1.00				1.00			
45-59	0.79	0.65	0.95	0.01	0.76	0.61	0.95	0.02
60-74	0.85	0.71	1.01	0.06	0.70	0.56	0.86	0.00
≥75	0.83	0.70	0.99	0.03	0.63	0.51	0.78	0.00
**Nephropathy type**								
Slowly progressive nephropathy	1.00				1.00			
Acute nephropathy	1.71	1.50	1.96	0.00	1.20	1.03	1.41	0.02
Variable/Uncertain progression	1.38	1.23	1.54	0.00	1.19	1.05	1.35	0.01
**Serum albumin**								
≥30 g/l	1.00				1.00			
<30 g/l	2.29	2.05	2.56	0.00	1.54	1.36	1.74	0.00
**Hemoglobin <10 g/dl**								
10-12 g/dl	1.00				1.00			
<10 g/dl	2.00	1.79	2.24	0.00	1.48	1.31	1.68	0.00
≥12 g/dl	0.84	0.71	1.01	0.06	0.90	0.75	1.09	0.28
**Number of cardiovascular diseases**								
0	1.00				1.00			
1	1.21	1.08	1.36	0.00	1.24	1.09	1.43	0.00
2	1.50	1.32	1.71	0.00	1.55	1.33	1.82	0.00
≥3	1.88	1.65	2.15	0.00	1.92	1.63	2.27	0.00
**Diabetes**								
No	1.00				1.00			
Yes	1.12	1.02	1.23	0.02	1.21	1.09	1.35	0.00
**Chronic respiratory disease**								
No	1.00				1.00			
Yes	1.42	1.25	1.62	0.00	1.23	1.06	1.43	0.01
**Active malignancy**								
No	1.00				1.00			
Yes	1.50	1.30	1.72	0.00	1.22	1.04	1.43	0.02
**Hepatic disease**								
No	1.00							
Yes	1.21	0.92	1.59	0.18				
**Mobility**								
Totally dependent	1.00				1.00			
Need assistance	1.85	1.59	2.15	0.00	1.33	1.13	1.57	0.00
Walk without help	2.55	2.05	3.18	0.00	1.71	1.36	2.16	0.00
**GP follow-up**								
At least 4 times per semester	1.00				1.00			
At least once per semester	1.01	0.90	1.13	0.87	1.06	0.94	1.21	0.35
Irregular follow-up	1.21	1.06	1.38	0.01	1.06	0.90	1.25	0.48

No follow-up	1.77	1.43	2.18	0.00	0.79	0.60	1.03	0.09
**Nephrologist follow-up**								
At least once every semester	1.00				1.00			
Irregular follow-up	1.91	1.71	2.14	0.00	1.32	1.17	1.50	0.00
No follow-up	4.55	4.05	5.11	0.00	1.83	1.58	2.12	0.00
**Creatinine measurement**								
At least one test per semester	1.00		1.00					
Gap of at least one semester after one test	1.93	1.70	2.20	0.00	1.24	1.07	1.45	0.00
At least one test only in the last 3 months before dialysis	2.74	2.25	3.33	0.00	1.43	1.13	1.82	0.00
No testing	4.52	3.53	5.79	0.00	1.68	1.23	2.31	0.00
**Number of hospital stays (<24h) ***								
0	1.00							
1	0.77	0.69	0.87	0.00				
≥2	0.71	0.64	0.80	0.00				
**Number of hospital stays (≥24h) related to nephrology**								
0	1.00				1.00			
1	0.70	0.62	0.79	0.00	0.83	0.72	0.95	0.01
≥2	0.82	0.74	0.91	0.00	0.96	0.85	1.09	0.55
**Number of hospital stays (≥24h) unrelated to nephrology**								
0	1.00							
1	1.18	1.05	1.33	0.00				
≥2	1.58	1.40	1.79	0.00				
**Days spent in hospital**								
0	1.00							
≥ 1 and ≤10	0.40	0.35	0.46	0.00				
≥11 and ≤31	0.55	0.48	0.63	0.00				
>31	0.79	0.68	0.91	0.00				
**RRT preparation type**								
Fistula or peritoneal dialysis catheter	1.00				1.00			
Other dialysis preparation care	4.69	3.89	5.65	0.00	3.47	2.86	4.23	0.00
No preparation	5.96	5.36	6.63	0.00	3.92	3.48	4.42	0.00

* Pooled Odds-Ratio and confidence intervals
